# The Importance of Free Fatty Chain Length on the Lipid Organization in the Long Periodicity Phase

**DOI:** 10.3390/ijms22073679

**Published:** 2021-04-01

**Authors:** Charlotte M. Beddoes, Denise E. Rensen, Gert S. Gooris, Marc Malfois, Joke A. Bouwstra

**Affiliations:** 1 Division of BioTherapeutics, Leiden Academic Centre for Drug Research, University of Leiden, 2311 EZ Leiden, The Netherlands; c.m.beddoes@lacdr.leidenuniv.nl (C.M.B.); d.e.rensen@umail.leidenuniv.nl (D.E.R.); gooris_g@lacdr.leidenuniv.nl (G.S.G.); 2 ALBA Synchrotron, Carrer de la Llum 2-26, 08290 Cerdanyola del Vallès, Barcelona, Spain; mmalfois@cells.es

**Keywords:** skin, stratum corneum, lipids, chain length, X-ray scattering, spectroscopy, permeability

## Abstract

The skin’s barrier ability is an essential function for terrestrial survival, which is controlled by intercellular lipids within the stratum corneum (SC) layer. In this barrier, free fatty acids (FFAs) are an important lipid class. As seen in inflammatory skin diseases, when the lipid chain length is reduced, a reduction in the barrier’s performance is observed. In this study, we have investigated the contributing effects of various FFA chain lengths on the lamellar phase, lateral packing. The repeat distance of the lamellar phase increased with FFA chain length (C20–C28), while shorter FFAs (C16 to C18) had the opposite behaviour. While the lateral packing was affected, the orthorhombic to hexagonal to fluid phase transitions were not affected by the FFA chain length. Porcine SC lipid composition mimicking model was then used to investigate the proportional effect of shorter FFA C16, up to 50% content of the total FFA mixture. At this level, no difference in the overall lamellar phases and lateral packing was observed, while a significant increase in the water permeability was detected. Our results demonstrate a FFA C16 threshold that must be exceeded before the structure and barrier function of the long periodicity phase (LPP) is affected. These results are important to understand the lipid behaviour in this unique LPP structure as well as for the understanding, treatment, and development of inflammatory skin conditions.

## 1. Introduction

The skin has the task of protecting the body from external influences and assisting in maintaining a homeostatic environment within the body [1]. It is the outermost layer of the skin, the stratum corneum (SC), which is charged with maintaining this barrier. This layer is comprised of highly impermeable keratin-filled corneocytes, encased in a well-defined extracellular lipid matrix [2]. This lipid matrix is the only continuous structure through the SC layer and is the preferred pathway for materials to transverse through this barrier. Thus, an understanding of the contributions of each lipid in this matrix is imperative to maintain the barrier’s effectiveness. 

The lipid matrix is comprised of three major lipid classes, ceramides (CERs), cholesterol (CHOL), and free fatty acids (FFAs), at approximately equimolar ratios. These extracellular lipids form two distinct co-existing crystalline lamellar structures with repeat distances of approximately 13 and 6 nm, which are referred to as the long and short periodicity phases (LPP and SPP, respectively) [3,4]. Within these lamellar structures, the majority of the SC lipids adopt a highly dense orthorhombic lateral phase, with a small proportion in the hexagonal or fluid phase [5,6]. Both the lamellar and lateral packing of the lipids are critical for barrier function and are controlled by the lipid composition. The composition of the SC lipid matrix is important for its barrier function. CERs and CHOL can form the LPP [7,8,9,10]; however, FFAs are required for an optimal orthorhombic lateral packing [11].

One of the common symptoms of many inflammatory skin diseases is a reduced skin barrier function. With these diseases, changes to the lipid matrix’s composition and arrangement are observed, which may explain the reduced barrier capability [12,13]. These alterations have far-reaching effects since worldwide, skin diseases are one of the most common health problems and the fourth leading cause of non-fatal disease burden when considering both dermatological and socioeconomic burdens [14]. Historically when considering the lipids, CER composition has been the major focus when investigating skin diseases. However, recent observations have identified that alterations in the FFA content can disrupt the barrier function just as much as CERs [15,16]. The most abundant FFA chain length found in the SC are C22, C24, and C26 [17]; however, concentrations of FFA C16 and C18 increase and the concentration of FFA C24 and C26 reduces in atopic eczema patients [18], while the average chain length also decreases in Netherton syndrome patients due to an abundance of FFA C20 and a larger reduction in C24 and C26 [19]. Previous investigations have compared the behaviour of non-LPP SC relevant lipid lamellae typically by shortening all of the FFA chains from C24 to C16 or C18 [16,20,21].

Therefore in this work, we have studied the effect of FFA chain length between the lengths of C16 and C28. We have studied how each of the major FFA chain lengths, observed in both healthy and diseased skin, affect the lipid organization and barrier function with the use of LPP lipid models. The LPP and its mainly orthorhombic lateral packing are crucial aspects for the skin barrier, and as a result, is the focus of this study. Lipid matrix models are models that are capable of closely resembling the structural and barrier behaviour as found in native SC. We show that the alterations in the LPP behaviour are not linear with chain length and that shorter FFA chain length models exhibit a different behaviour compared to their longer chained counterparts. Similar behaviour is also observed with the lateral packing, only models containing FFAs C20 and longer have the orthorhombic phase transition temperature measured to increase with increasing chain length, which may be explained by the phase separation of the shorter FFAs. We compare the phase separation behaviour between the LPP models with different lipid compositions. We also show that the LPP is a robust structure, and can withstand a certain threshold of change before its barrier function is affected. The significance of our results in inflammatory skin diseases is discussed.

## 2. Materials and Methods

### 2.1. Materials

Esterified omega-hydroxyacyl-sphingosine (CER EOS), as well as shorter CERs including: N-(tetracosanoyl)-sphingosine (CER NS), N-(tetracosanoyl)-phytosphingosine (CER NP); N-(2R-hydroxy-tetracosanoyl)-sphingosine (CER AS), and N-(2R-hydroxy-tetracosanoyl)-phytosphingosine (CER AP), were all kindly donated by Evonik, Essen, Germany. CER nomenclature was used based on the definitions from Motta et al. [22]. The sphingoid chain length for all the CERs was C18, while the acyl chain was C24, except for CER EOS with C30 chain, and an additional shorter CER NP with C16 acyl chain. Palmitic acid (C16), stearic acid (C18), arachidic acid (C20), behenic acid (C22), tricosylic acid (C23), lignoceric acid (C24), cerotic acid (C26), octacosanoic acid (C28), and cholesterol (CHOL) were purchased from Sigma-Aldrich Chemie GmbH, Schnelldorf, Germany. The deuterated FFA C16 and C24, which were deuterated along the entire acyl chain (C16-d31 and C24-d47), were obtained from Larodan (Malmö, Sweden) and Arc Laboratories B.V. (Apeldoorn, The Netherlands), respectively. All solvents used were of analytical grade and supplied by Labscan, Dublin, Ireland. The water was of Millipore quality produced by a Milli-Q water filtration system with a resistivity of 18 MΩ cm at 25 °C. Nuclepore polycarbonate filters, with 0.05 μm pore size were purchased from Whatman, (Kent, UK).

### 2.2. Lipid Matrix Models

Two different models were prepared with synthetic lipids, with the ratio among CERs, CHOL, and FFAs remain in an equimolar ratio; however, the composition of the lipid subclasses was changed. In the simplistic model, the lipid composition was comprised of CER EOS and NS in a 40:60 molar ratio, CHOL, and a single-chain length FFA (C16–C24) to monitor the behavioural changes induced by the various FFA chain lengths. In addition, a porcine SC lipid composition mimicking model was used to determine the adaptability of the LPP. The CERs subclasses consisted of CER EOS (C30): CER NS: CER NP: CER AS: CER NP (C16): CER AP in a molar ratio of 40:36:11:3:6:4, a similar composition found in porcine SC [8]. The FFA ratio in this model consisted of 1.8:4:7.7:42.6:5.2:34.7:4.19 for the saturated FFA C16: C18: C20: C22: C23: C24: C26, a composition similar to native SC [23], after which the FFA composition was changed to increase the content of FFA C16, while maintaining the ratio of the remaining FFAs. Regardless of the model used, CER EOS concentration was increased from native concentrations (12%) to 40 mol% of the CER content, to ensure the LPP would form exclusively and to exclude overlapping with the CHOL peak [24,25]. This elevation does not affect the LPP structure [24]. For the partially deuterated Fourier-transform infrared spectroscopy (FTIR) measurements, FFA C24 and C16 were substituted with either FFA C24-d47 or C16-d31.

### 2.3. Small Angle X-ray Diffraction (SAXD)

The SAXD measurements were performed at the European Synchrotron Radiation Facility (ESRF, Grenoble, France) at station BM26B, and the NCD-SWEET beamline at the ALBA synchrotron (Barcelona, Spain). For measurements at the ESRF, 0.75 mg of lipid at the desired composition was prepared in 2:1 hexane/ethanol solvent at a concentration of 4.5 mg/mL. The mixture was sprayed using a y-axis adapted Camag Linomat IV sample applicator (Muttenz, Switzerland) on a nuclepore polycarbonate filter, under a steady flow of nitrogen, over an area of 1 cm^2^. Once sprayed, the models were heated until melted (85 °C) and equilibrated for 30 min. Once cooled, samples were hydrated for ≥15 h in a humid environment (pH 5.0). Before the measurement, a 2 × 5 mm^2^ strip of the sample was loaded into the beam. At the ESRF the SAXD images were generated using a Pilatus 1M detector with 981 × 1043 pixels of 172 μm^2^ spatial resolution. The calibration of the detectors was performed using silver behenate. For the diffraction measurements, the X-ray wavelength was set at 1.033 Å and the detector-to-sample distance was 2.1 m. The samples were measured for 120 s, at 25 °C. The number of repeats (*n*) for each condition was ≥2. The two-dimensional (2D) scattering was integrated over a 40° segment perpendicular to the orientation of the sample, of which the segment starting point was located at the beam centre.

Samples for measuring at ALBA were prepared using a similar method; however, 1 mg of lipids was sprayed over an area of 1 × 3 mm, and before measuring, samples were hydrated in an oxygen-free, 84% relative humidity environment for 5 days at room temperature. At ALBA, the wavelength was set at 0.999 Å, and the Pilatus 1M detector was set at a distance of 2.148 m from the sample. The experimental set up was calibrated with silver behenate. The samples were measured for 20 s at 23 °C. The one-dimensional intensity profiles were calculated by integrating starting from the beam centre, over a 90° segment. Samples were measured *n* = 2.

The 1D intensity profiles were calculated using the Bragg equation. Peak position was fitted with Fityk [26], and the repeat distance periodicity (*d*) was calculated using least square fitting and the equation: *d* = *2hπ*/*q*, in which *h* is the order number of the diffraction peak at a certain position (*q*).

### 2.4. FTIR Measurements

The sample preparation method for the FTIR measurements was similar as described for the SAXD measurements. 1.0 mg of lipid at the desired compositions was dissolved in 2:1 chloroform/methanol (CHCl_3_/MeOH), at a concentration of 5 mg/mL. The mixture was sprayed on an AgBr window over an area of 1 cm^2^. Once sprayed, the model was equilibrated to 85 °C for 30 min and cooled. The lipids were then hydrated in deuterated acetate buffer (pH 5.0) at 37 °C for ≥ 15 h, to ensure the sample had sufficient time to fully hydrate.

FTIR spectroscopy measurements were performed using the Frontier FTIR spectrometer (Perkin Elmer, Buckinghamshire, UK), equipped with a broad-band mercury cadmium telluride detector. ≥10 min before and during measuring, the sample compartment was purged with dry air. The lipid models were measured between 0 °C and 90 °C, at a rate of 0.25 °C/min, at a 1 cm^−1^ resolution. The data were collected in steps of 4 min in absorbance mode. The spectra of the temperature run were collected using the software Spectrum TimeBase (PerkinElmer). Measurements were performed at *n* ≥ 2.

The peak position of the CH_2_ and CD_2_ symmetric stretching vibration (ν_s_CH_2,_ 2845–2855 cm^−1^, and ν_s_CD_2,_ 2080–2100 cm^−1^) was determined by the centre of gravity (CoG) method. The peak height for CoG was set between 80 and 90%. The lateral phase transition temperatures were determined by the linear regression fit of the peak shift of the ν_s_CH_2_ and ν_s_CD_2_, and by the CH_2_ and CD_2_ scissoring vibrational peak splitting distance (δCH_2,_ 1462–1473 cm^−1^, and δ_s_CD_2,_ 1080–1100 cm^−1^). The spectra were deconvoluted using the Spectrum (PerkinElmer) software and plotted with in-house Enthought Canopy scripts. The ratio between orthorhombic and hexagonal lateral packing was calculated by first fitting the δCH_2_ peaks with a Pearson7 function in Fityk, then by dividing the height of the second orthorhombic phase peak (1475 cm^−1^) from the hexagonal peak (1472 cm^−1^).

### 2.5. Trans Epidermal Water Loss (TEWL) Measurements

The samples were prepared in a similar way as described for the X-ray samples. A total of 0.9 mg of lipids were dissolved in hexane/ethanol (2:1) solution, to a concentration of 4.5 mg/mL. The lipids were sprayed on the porous filter (Whatman Nuclepore Track-Etch Membrane), under a steady flow of nitrogen, over an area of 1 cm^2^. Samples were equilibrated at 85 °C for 30 min before cooling back to room temperature. Once equilibrated the sample was loaded into a PermeGear in-line diffusion cell (Bethlehem, Pennsylvania, USA), which was filled with Milli-Q water and left to hydrate for 30 min before measuring.

TEWL measurements were performed using an AquaFlux (model no. AF201502, Biox Systems Ltd.) and the software BioFlux, recording for a total of 30 min with 10 sec intervals. The average steady-state flux (J) for each sample was determined as the average flux value measured during the final 3 min of the measurement, *n* ≥ 4. One-way ANOVA with Dunnett multiple comparison test was performed to analyse the TEWL data. The significance level for rejection of the null hypothesis was set at *p* < 0.05 (*), *p* < 0.01 (**), *p* < 0.001 (***), and *p* < 0.0001 (****).

## 3. Results

### 3.1. Behavioural Changes Due to Increasingly Shorter FFAs Are Not Categorically Linear

**Figure 1:**
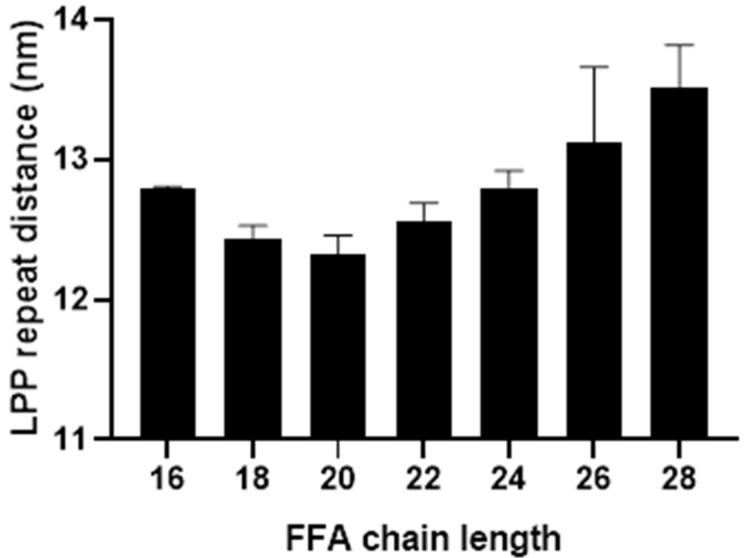
The average repeat distance of the long periodicity phase (LPP) in the simple model with free fatty acid (FFA) C16–28. Between C20 and 28, the LPP repeat distance increases as the chain length increases, while at C16–18 the LPP repeat distance increases as the FFA chain length decreased. The LPP repeat distances for the models containing C16, C18, C20, C22, C24, C26, and C28 were 12.8 ± 0.02, 12.4 ± 0.1, 12.3 ± 0.1, 12.6 ± 0.1, 12.8 ± 0.1, and 13.1 ± 0.5, respectively. Error is calculated as the standard deviation between repeat measurements.

The effect of the FFA chain length on the LPP repeat distance was identified with SAXD measurements. Simple models forming the LPP were prepared with a single FFA chain length varying between C16 and C28. The full 1D diffraction profiles at each FFA chain length can be found in Figure S1. Figure 1 presents the repeat distances of the LPP against the FFA chain length. From this figure, it is observed that with decreasing FFA chain length the LPP repeat distance also decreases; however, this is only true for models containing a FFA length varying between C20 and C28. Below chain lengths of C20, as the FFA chain length decreased, the LPP repeat distance increases, implying a difference in the model’s behaviour. In addition, the 1D curves of the shorter FFA models show a small additional peak that varied in length between 4.5 and 6.0 nm (Figure S1), no further orders were observed and, therefore, this phase could not be identified further.

To further investigate the alterations that are occurring within the LPP due to the FFA chain length variation, the lateral packing of the lipids was studied using FTIR. Information on the conformational ordering and lipid packing is obtained from the position of ν_s_CH_2_, and the splitting behaviour of δCH_2_. The highly ordered orthorhombic phase is associated with a high conformational ordering that is when the ν_s_CH_2_ peak is located at ~≤2850 cm^−1^. As the lipid lateral packing changes from an orthorhombic to hexagonal phase the peak position shifts by ~1 cm^−1^, and further increases by ~2.5 cm^−1^ when the lipids change from a hexagonal to a fluid phase (Figure S2). The orthorhombic to hexagonal transition was only measurable in samples between FFA C20 and C28 samples since the hexagonal packing of the FFA C16 and C18 models are already prominently present at low temperatures. The mid transition temperatures for the orthorhombic to hexagonal phase for the C22, C24, C26, and C28 models were 32.1 ± 2.5, 39.2 ± 0.5, 43.0 ± 2.0, and 41.8 ± 1.2 °C respectively, while the hexagonal to fluid phase transition values did not follow a trend with FFA chain length, with mid-transition temperatures of 72.1 ± 2.7, 74.6 ± 0.6, 72.5 ± 6.6, 67.4 ± 2.5, 72.7 ± 7.7, 65.1 ± 5.5, and 69.4 ± 1.9 for the C16, C18, C20, C22, C24, C26, and C28 models.

**Figure 2:**
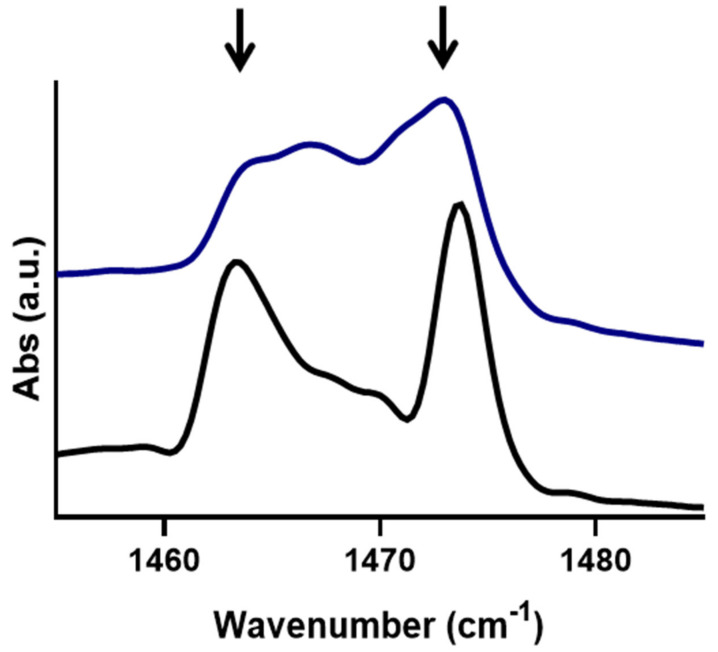
The δCH_2_ splitting effects when containing either FFA C24 (black) or FFA C16 (blue) at 10 °C. Black arrows indicate the position of the orthorhombic peak.

To identify the difference in the domain size and lipid population in the orthorhombic phase, the shape and splitting distance of the δCH_2_ peaks were compared. The δCH_2_ peaks of the models with FFA C24 and C16 at 10 °C are presented in Figure 2. Both models have a proportion of the lipids packed in an orthorhombic lateral packing, as indicated by the presence of the peaks at 1464 and 1475 cm^−1^ (Figure 2, black arrows). When comparing the splitting lengths, the C24 and C16 models had a length of 10.3 and 6.3 cm^−1^ respectively. This shorter splitting observed in the C16 model implies that the lipids are arranged in smaller domains compared to the C24 model. Figure 2 also shows that the orthorhombic peaks in the C24 model are more defined, and the hexagonal peak at 1467 cm^−1^ is much smaller than in the C16 models. The height ratio between the 1467 cm^−1^ hexagonal and 1475 cm^−1^ orthorhombic peaks showed that the proportion of hexagonal packing increased from 0.32 in the C24 model to 0.73 in the C16 model.

**Figure 3:**
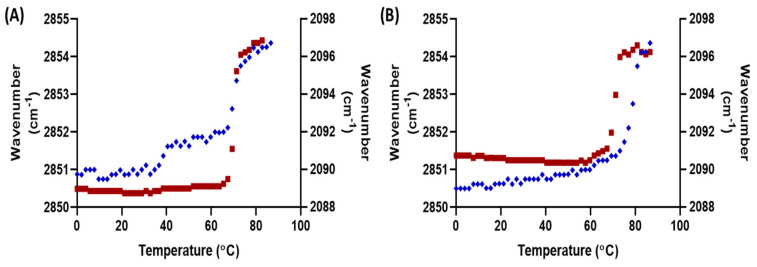
Thermotropic response of ν_s_CH_2_ (blue) and ν_s_CD_2_ (red) of the (**A**) FFA C24 and (**B**) C16 models. The CH_2_ and the CD_2_ peak positions are plotted on the primary and secondary y-axis’s, respectively, displaying the phase transition temperatures of the ceramide (CER) and dFFA chains in the lipid models. All models contain CERs EOS and NS at a ratio of 40:60. Overall, the composition of CERs, cholesterol (CHOL), and FFAs is in an equimolar ratio.

The mixing behaviour of FFA C16 and C24 within the membrane models were observed by incorporating their deuterated counterparts. The ν_s_CD_2_ describes the conformational ordering of the deuterated FFAs. Figure 3 shows the orthorhombic to hexagonal and hexagonal to fluid transition temperatures of the ν_s_CH_2_ and ν_s_CD_2_. In the deuterated FFA (dFFA) containing C24 model, the fluid mid transition temperature was similar for the protiated and deuterated lipids, occurring at 71 and 70 ± 0.0 °C, respectively, implying the dFFAs are mixed with the protiated lipids. In contrast, the mid hexagonal to fluid phase transition of the ν_s_CH_2_ and ν_s_CD_2_ in the dFFA C16 model, has a difference of ~10 °C (78.9 ± 0.6 and 69.0 ± 3.1 °C respectively), thus implying that a portion of the FFA C16 has phase separated from the LPP. Deuterated FFA C16 has a melting temperature of 61–64 °C; however, the phase-separated lipids in the C16 model have a higher melting temperature of 68–71 °C; this shift in the melting temperature implies it is not purely FFA C16 that has separated. The SAXD curves do show an additional phase in the shorter C16 model; thus, a proportion of C16 FFAs that had phase-separated may have incorporated themselves into the structure responsible for the additional peak. However, it is unlikely to be pure FFA C16 since it has a repeat distance of 3.5 nm (Figure S3), while our results showed the phase-separated structure in the C16 model to have a repeat distance of 4.5 nm (Figure S1), a difference of 1 nm. This suggests that additional lipids had also phase separated together with the FFA C16. The mid orthorhombic to hexagonal phase transition temperature could not be identified for the dFFA C16 model (Figure 3B).

**Figure 4:**
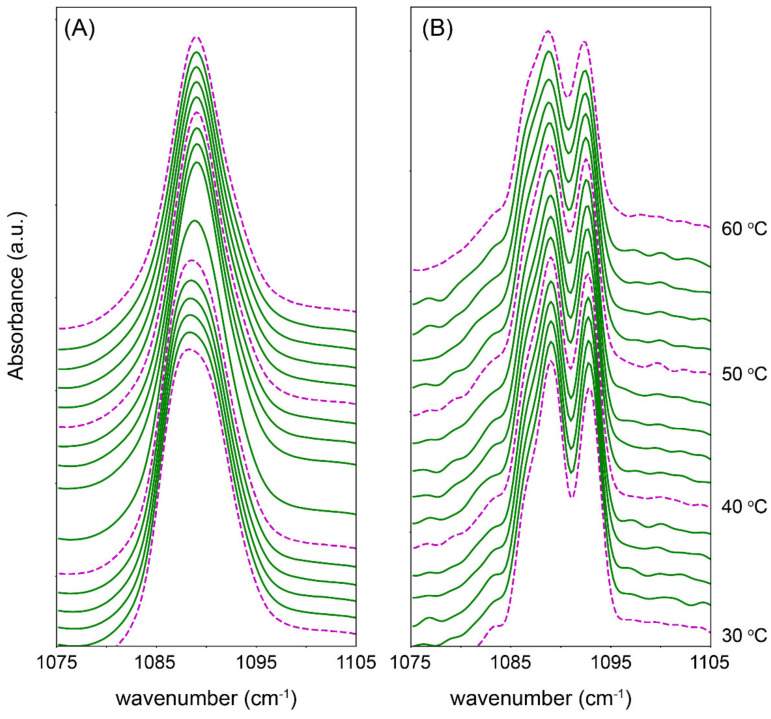
δCD_2_ of the (**A**) FFA C24 and (**B**) FFA C16 models, between 30 °C and 60 °C. Every 10 °C is highlighted by a dotted maroon curve. All models contain CERs EOS and NS at a ratio of 40:60. Overall, the composition of CERs, CHOL, and FFAs are in an equimolar ratio.

Further details on the inter-lipid interactions can be identified by the splitting and broadening of the δCH_2_ and δCD_2_ peaks. When the deuterated FFA chains are mixed with the protiated chains the δCH_2_ and δCD_2_ vibrational energy is different enough that the short range coupling responsible for the peak splitting when in the orthorhombic phase is no longer possible. Using the same FFA deuterated models depicted in Figure 3, the δCD_2_ curves are shown in Figure 4. At 30 °C, the splitting in the spectrum of the C24 model does not occur, and instead appears as a broad single peak (Figure 4A), implying the deuterated FFA C24 is mixed with the protiated chains. As the lipids are heated to 44 °C, the peak becomes sharper, indicating the loss of all short range interactions as the lipids pack in a purely hexagonal lattice. Figure 4B shows that C16 exhibited short distant splitting, demonstrating that FFA C16 had phase separated and were neighbouring each other in an orthorhombic phase. The orthorhombic packing only disappeared once the lipids melt into the fluid phase at 76 °C. When combining these results with the observed phase transition temperature shift in Figure 3, and the remaining orthorhombic domain at 60 °C in Figure 2, it can be concluded that the sample is in a mixed-phase. In this mixed phase, the phase separated FFA C16 rich structure remains in an orthorhombic phase at higher temperatures, while the remaining lipids are in a hexagonal phase. This configuration would explain the rapid hexagonal to fluid phase transition, and at lower temperatures the steady rise in wavenumber with temperature as seen in Figure 3. This is further supported by the observed direct transition from orthorhombic to fluid packing, omitting the transition to the hexagonal phase of the FFA C16 separated lipids. This orthorhombic to fluid phase transition is typical pure FFA thermotropic behaviour [27].

Our results highlight that the FFA’s effect on the LPP depends on whether the FFA chain length in the model is longer (C20–C28) or shorter (C16–C18). The difference in behaviour between shorter and longer FFAs may be explained due to the hydrophobic mismatching between the FFA and CER chains. Previously it has been shown that FFA C24 is located throughout the length of the LPP [28], and in the case of FFA C24, it is located in the same regions as the acyl chains of the CERs [28,29]. In our model and the majority of CERs in native SC, the CERs have longer acyl carbon chains (C24), which match well with the longer FFA chain lengths. However, for the shorter FFA models (C16 and C18), neighbouring with the longer CER C24 acyl chains causes hydrophobic mismatching and may introduce elastic stress upon the lipid lamellae [30]. Once the amount of stress exceeds the level that the lamellae can accommodate, a rearrangement of the lipids is required to relieve the stress. One method available is for the lipids to phase separate, and form energetically more favourable structures.

### 3.2. An Optimal Barrier Function Is Possible with Increased Shorter FFAs Content, within a Finite Range

Throughout our lifecycle, the lipid composition within the SC changes due to factors such as age and seasonal influences [31,32]; thus, the LPP has naturally evolved to withstand small alterations with minimal effect on the barrier function. One method that assists with the cohesion of the lipids is a distribution of the lipid chain lengths [16,33]. However, to maintain an optimal barrier function, the deviation of chain length distribution is finite. Divergence from the FFA chain length composition, to the extent of influencing the barrier function, is seen in inflammatory skin diseases, such as atopic eczema [18], and Netherton syndrome patients [19]. Although substituting all the FFA chain lengths from one length to another can provide information on the overall effect, it is not realistic since only partial substitution is observed in skin diseases.

To determine how relevant the changes in the LPP and its lateral packing are to the barrier function, the compositional adaptability before a structural change or reduction in barrier performance was determined. A lipid model that mimicked the composition of porcine SC was used. This composition is composed of readily available synthetic lipids, which can mimic the barrier’s ability, and structural properties of human SC [8,23]. The lamellar phase behaviour was monitored as the amount of FFA C16 was altered in relation to the total FFA content, while the other remaining FFAs remained at the same ratio. The LPP repeat distances for the models containing 1.8, 20, 30, 40, and 50% FFA C16 were 12.7 ± 0.18, 12.8 ± 0.02, 12.9 ± 0.1, 12.7 ± 0.03, and 12.6 ± 0.19, respectively (Figure S5). At FFA C16 concentrations of 40% and higher, an additional peak with a repeat distance of 3.6 nm is present, as well as a broadening and asymmetry of the third peak, implying that phase separation has occurred. 3.6 nm is similar to the repeat distance measured for pure FFA C16 bilayer (Figure S4), supporting the notion that the separated material might be purely FFA C16, as seen in the earlier model.

**Figure 5:**
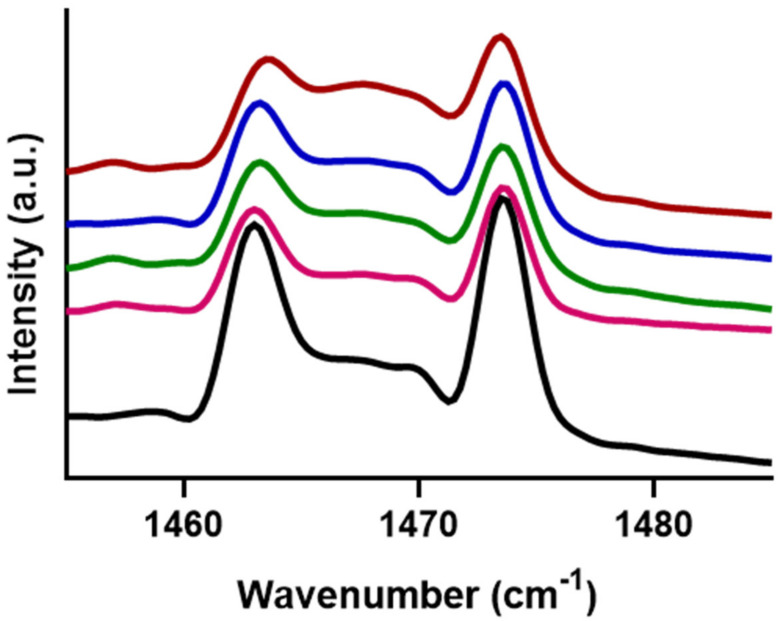
δCH_2_ for models containing FFA C16 content at 1.8 (black), 20 (magenta), 30 (green), 40 (blue), and 50 (red) percent of the total FFA content at 10 °C. Regardless of FFA C16 content, the splitting distance between the orthorhombic peaks does not change; however, the intensity of the central hexagonal peak at 1468 cm^−1^ increases with increasing C16 content.

To further probe the phase separation behaviour occurring at higher FFA C16 concentrations, lateral packing was monitored, at the same FFA C16 proportion. At all concentrations measured between 1.8 and 50% the ν_s_CH_2_ thermotropic curves were similar, with the orthorhombic to hexagonal mid-transition temperature for the 1.8, 20, 30, 40, and 50% being 34.6 ± 0.1, 33.0 ± 0.4, 30.6 ± 0.5, 33.5 ± 1.6, and 31.6 ± 1.5, and for hexagonal to fluid mid-phase transitions being 71.8 ± 0.8, 69.6 ± 2.8, 74.5 ± 0.5, 70.2 ± 2.0, and 68.8 ± 34 °C, respectively; an example of each of the individual curves is seen in Figure S6. The δCH_2_ in Figure 5 shows that the peak positions do not shift; thus, the splitting length remains at ~10 cm^−1^ regardless of the FFA C16 content, indicating the mean orthorhombic domain size did not change. However more noticeable is the increase in the intensity of the central hexagonal peak as the concentration of FFA C16 increases in the models. The increase in the height ratio of the hexagonal peak 1467 cm^−1^ compared to the orthorhombic peak at 1475 cm^−1^ from 0.29 in the 1.8% model to 0.67 in the 50% model, suggests a significant increase in the proportion of lipids packed in the hexagonal phase.

**Figure 6:**
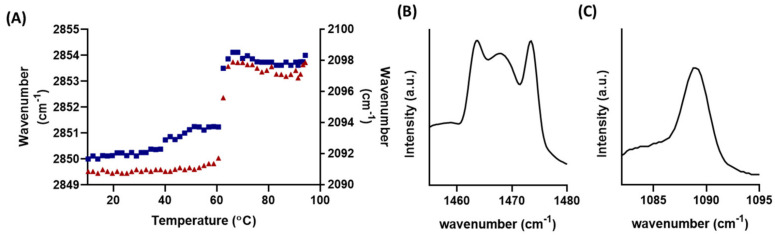
(**A**) Thermotropic response curves of ν_s_CH_2_ (blue) and ν_s_CD_2_ (red) of the 50% FFA C16 model. The CH_2_ and the CD_2_ peak positions are plotted on the primary and secondary y-axes respectively, displaying the phase transition temperatures of the protiated and dFFA C16 chains in the lipid models. (**B**) δCH_2_ and (**C**) δCD_2_ of the FFA C16 50% model, at 25 °C. The composition of CERs, CHOL and FFAs are in an equimolar ratio.

To try and identify the phase-separated lipid composition, the mixing behaviour of FFA C16 was monitored. The phase separation was most prominent when FFA C16 concentration was at 50% (Figure S5), and this composition was used to further study the phase-separated structure using deuterated FFA C16. Figure 6A shows that the protiated lipids and the deuterated FFA C16 melted in the same temperature range; however, this coincidentally is also the melting temperature of pure dFFA C16, so from these data alone, it is not possible to determine if the FFA C16 is mixed with the other lipids or phase separated. When combined with the δCH_2_ and δCD_2_ peaks in Figure 6B,C the δCH_2_ curve shows a disruptive orthorhombic splitting at 24 °C. Figure 5 implies a small proportion may originate from hexagonal packing; however, due to the intensity of the peak in Figure 6B it is likely to be additionally supplemented by interactions with deuterated chains neighbouring the protiated chains, and thus indicating that these lipids are mixed. This is further supported by the δCD_2_ vibration in Figure 6C that shows a single peak, demonstrating the absence of interactions between the deuterated chains. Their mixing with the protiated chains prevents the splitting phenomena that occurred in the orthorhombic phase, thus further supporting the assumption that the FFA C16 has not phase separated exclusively in these models.

Based on the short repeat distance of the phase separated structure, another lipid with a similar length is CER NP (C16). It is not possible to investigate if this lipid had phase separated with FTIR since deuterated CER NP (C16) is currently not commercially available. Alternatively, the phase separation may be due to a small proportion of CER NP (C24). Phase separated crystalline NP has been previously reported to locate at q = 1.71 nm^−1^ (d = 3.67 nm), a similar length found in our curves [10]. CER NP has been previously proposed to pack in a V-shape conformation, with a repeat distance of 3.73 nm [34]. It is also possible that a mixture of CER NP (C24): CHOL: FFA C16 could have phase-separated; this also forms a structure at around 3.7 nm; however, it also forms additional structures making the 1D profile very noisy (Figure S7), which is not observed in our X-ray curves, implying this is not what is occurring in our samples.

**Figure 7:**
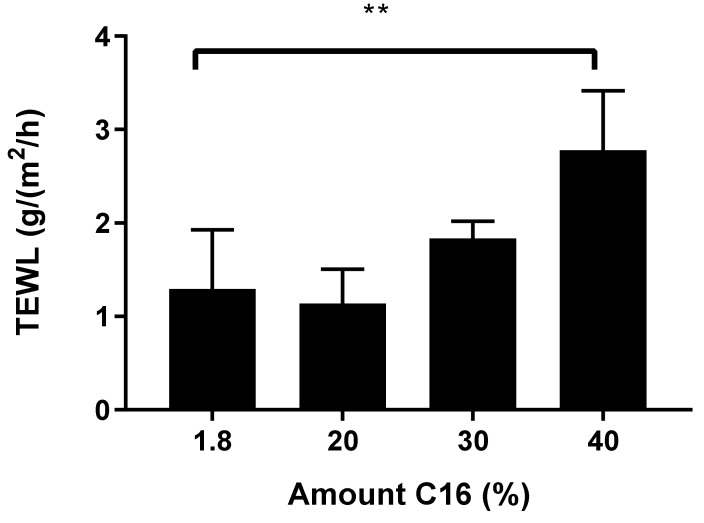
The steady-state flux values from TEWL measurements across the model membranes with different percentages of FFA C16 content in relation to the total FFA content. When the FFA C16 was increased from 1.8 to 40%, the flux was statistically higher, *n* = 4 ** *p* < 0.01.

Regardless of the physical alterations, knowledge of barrier properties is also important to understand due to increasing concentrations of FFA C16; thus, the effect on the inside-outside barrier function was measured with TEWL. The same modified porcine lipid models were measured as described previously. The average steady-state fluxes are presented in Figure 7. The 1.8% model, which mimics the FFA composition found in normal SC, has a steady-state flux of 1.3 ± 0.6 g/m^2^/h. A similar flux value is measured at 1.1 ± 0.4 g/m^2^/h when the FFAC16 content was increased to 20%. As the FFA C16 content increased to 30 and 40%, a higher steady-state flux value was measured at 1.8 ± 0.2 (not statistically different) and 2.8 ± 0.6 g/m^2^/h, respectively (statistically significantly different compared to 1.8%).

The poorer barrier ability found in models with an increasing proportion of shorter FFA may be explained by the increase in overall conformational disordering, arising from these shorter FFAs. The ν_s_CH_2_ and ν_s_CD_2_ wavenumber values represent the packing density of the lipid chains, with the higher the wavenumber, the lower the lipid chain packing density. This could be the result of an increasing proportion of the lipids shifting from the orthorhombic to hexagonal phase. In our FFA C16 50% models the lipids had a ν_s_CH_2_ and ν_s_CD_2_ wavenumber of 2850.1 and 2090.7 cm^−1^, respectively; these values are higher compared to the 1.8% SC lipid model with wavenumbers around 2846 and 2089.4 cm^−1^, respectively [35]. This increased conformational disordering together with the decreased packing density can explain the increase in TEWL values with increasing FFA C16 content. This effect is also observed within a similar SPP model [36]. A similar study investigated the effect of partial FFA chain shortening, using a human mimicking model containing both the LPP and SPP phases [15]. To mimic the different composition observed in atopic eczema patients compared to healthy individuals [18], the FFA ratio between the long (C24–20): short (C16–C18) was reduced from 94.2:5.8 to 45:55, resulting in a difference of ~50% of the total FFA content in the model. Due to this change, a significant increase in the TEWL and permeation of ethyl p-aminobenzoate was observed. In the same human model, the chain length of sphingosine based CERs was reduced from C24 to C16, replacing 13% of the total CER content [15]. At this level of substitution the permeability of ethyl-p-aminobenzoate and lateral lipid packing density did not significantly deviate from the healthy model; thus, expressing a similar behaviour due to chain length shortening, regardless of the difference in the lipid models.

The results of this study imply that FFA chain length does have an effect on the LPP structure and barrier function at inflammatory skin disease relevant concentrations. However, it is important to identify how significant these are in relation to other changes that are observed simultaneously in diseased skin. Aside from shortening the FFA chain length, other alterations commonly seen in inflammatory skin diseased SC include CER chain length shortening [37], CER headgroup substitutions [38,39,40], increased unsaturation of FFAs [18,19], and changes in the CER:CHOL:FFAs ratio [22], all leading to an increase in permeability. However, determining which has a greater contribution to the reduced barrier function remains a challenge. Uche et al. have investigated the effect of CER chain length shortening, CER headgroup substitution, FFA chain length shortening, and all of the conditions combined to mimic the alterations in atopic dermatitis patients [15]. After comparing the structure and barrier capabilities under these conditions, the largest reduction in the barrier was attributed to the change in CER and FFA carbon chain length, which in turn caused a decrease in the lipid packing density. In contrast, they found that alterations in the CERs affected the lamellar organization but not the overall barrier function. Thus, since the FFAs and CERs primarily affect different aspects of the models, it is not possible to focus on a single aspect as the main contributor to diseased SC, and FFAs should be equally studied in diseased studies.

## 4. Conclusions

In summary, our results show a non-uniform effect that the FFAs chain length has on the LPP in SC lipid models. The behavioural change observed depends on whether a mismatch with the remaining lipid chain is present. Modulations of the FFA C16 concentration in porcine mimicking lipid models showed a threshold concentration of 30% of total FFA content before the barrier performance and lateral packing density were reduced and phase separation occurred. This study expands on the current knowledge of the FFA chain length effect, and the stability the LPP structure in response to changes in FFA chain length before a decrease in its barrier performance is observed. These results are highly relevant for understanding the mechanisms governing the change in inflammatory skin diseases, as well as for the development of treatments for such alterations in the skin.

## Data Availability

Data are contained within the article or supplementary material.

## Supplementary Materials

The following are available online at https://www.mdpi.com/article/10.3390/ijms22073679/s1, Figure S1: SAXD curves for the simple model containing FFA chain lengths of (A) C16, (B) C18, (C) C20, (D) C22, (E) C24, (F) C26, and (G) C28. The Arabic numbers indicate the peak orders of the LPP. Crystalline CHOL peaks at 1.85 and 3.68 nm^−1^ are indicated by an asterisk (*). Unassigned peaks are highlighted with an arrow. CER:CHOL:FFA are in a molar ratio of 1:1:1 for all models.; Figure S2: Thermotropic response curves of the simple model with FFAs (A) C16, (B) C18, (C) C20, (D) C22, (E) C24, (F) C26, (G) C28. Error is calculated as the standard deviation. All models were prepared with CER:CHOL:FFA in a molar ratio of 1:1:1.; Figure S3: X-ray diffraction curve of FFA C16, lipids form a bilayer with a repeat distance of 3.6 nm; Figure S4: δCD_2_ of FFA C16 models, between 0 and 90 °C. Every 10 °C is highlighted by a dotted maroon curve. At low temperature the twin peaks or the orthorhombic phase at 1464 and 1475 cm^−1^, can be observed with the single hexagonal peak at 1472 cm^−1^ These peaks remain as the temperature increases, until 76 °C when the orthorhombic peaks are no longer present, and the lipids are melting. All models contain CERs EOS and NS at a ratio of 40:60. Overall the composition of CERs, CHOL, and FFAs are in an equimolar ratio; Figure S5: SAXD curves for the porcine model containing FFA C16 at ratios of (A) 1.8, (B) 20, (C) 30, (D) 40, and (E) 50%. The Arabic numbers indicate the peak orders of the LPP. Crystalline CHOL peaks at 1.85 and 3.68 nm^−1^ are indicated by asterisk (*). The phase separation peak is indicated with an hashtag (#) All models were prepared with CER:CHOL:FFA in a molar ratio of 1:1:1.; Figure S6: Thermotropic response curves of the porcine model with FFAs C16 content at (A)1.8, (B) 20, (C) 30 (D), 40 and (E) 50% of total FFA content. Orthorhombic to hexagonal and hexagonal to fluid transition temperatures for all samples were similar with an average mid transition temperature of 32.7 ± 1.6 and 71.0 ± 2.2 °C. All models were prepared with CER:CHOL:FFA in a molar ratio of 1:1:1.; Figure S7: X-ray diffraction curve of CER NP: CHOL: FFA C16 at a 1:1:1 molar ratio, the main repeat distance is 3.7 nm; however, additional structure at lower concentrations also form at this composition.
