# Supplementary material for: The Importance of Free Fatty Chain Length on the Lipid Organization in the Long Periodicity Phase
Source: Int J Mol Sci. 2021 Apr 1;22(7):3679. doi: 10.3390/ijms22073679 (PMC8038103; doi:10.3390/ijms22073679)
Supplement: Supplementary file 1 [file ijms-22-03679-s001.pdf]

Supplementary Materials

The importance of free fatty chain length on the lipid organization in the long periodicity phase

Charlotte M. Beddoes[1], Denise E. Rensen[1], Gert S. Gooris[1], Marc Malfois[2], Joke A. Bouwstra[1]

[1] Division of BioTherapeutics, Leiden Academic Centre for Drug Research, University of Leiden, Leiden, The Netherlands.

[2] ALBA Synchrotron,Carrer de la Llum 2-26, 08290 Cerdanyola del Vallès, Barcelona, Spain

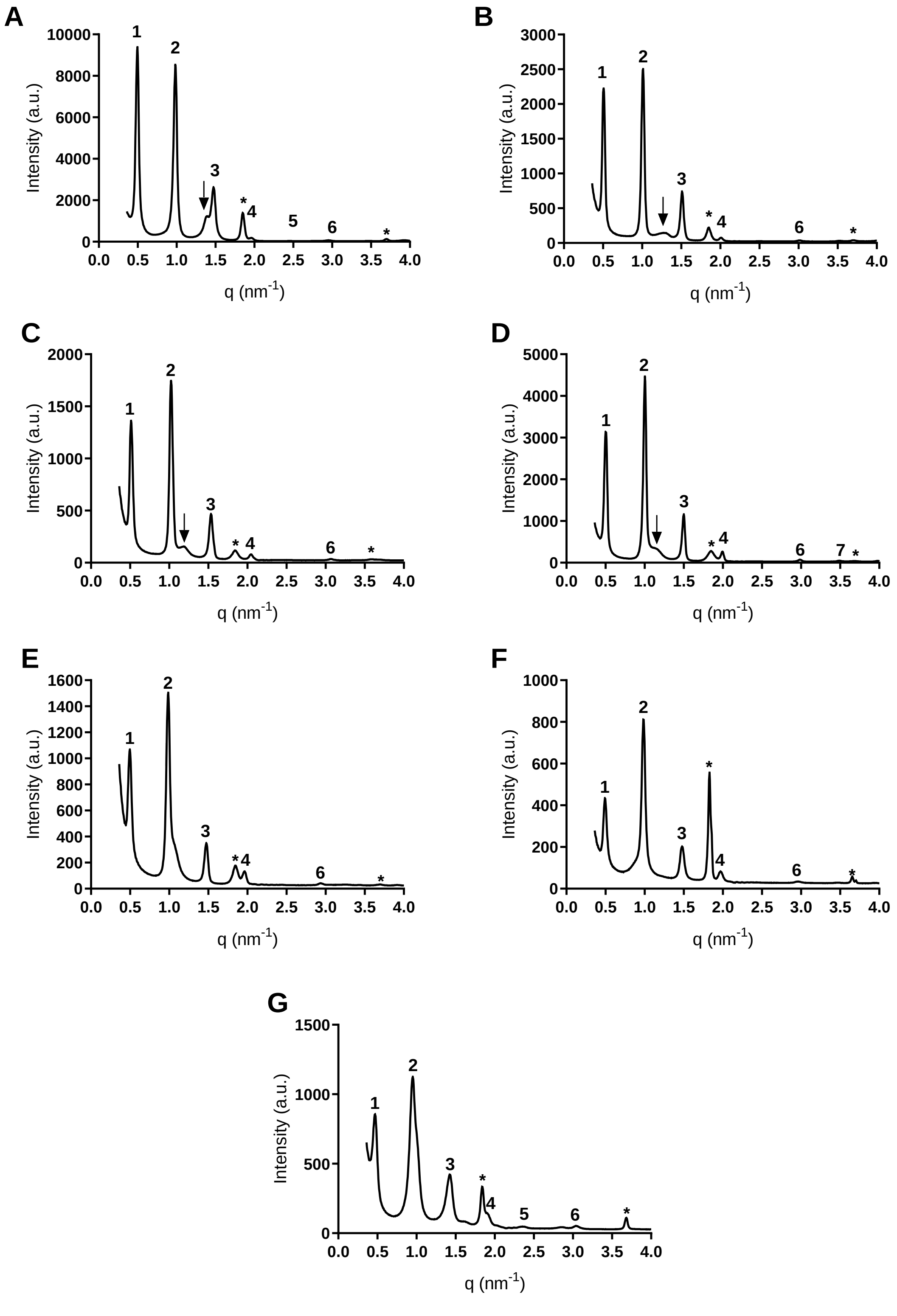

Figure S1: *SAXD curves for the simple model containing FFA chain lengths of (A) C16, (B) C18, (C) C20, (D) C22, (E) C24, (F) C26, and (G) C28. The Arabic numbers indicate the peak orders of the LPP. Crystalline CHOL peaks at 1.85 and 3.68* nm^{-1} *are indicated by an asterisk (*). Unassigned peaks are highlighted with an arrow. CER:CHOL:FFA are in a molar ratio of 1:1:1 for all models.*

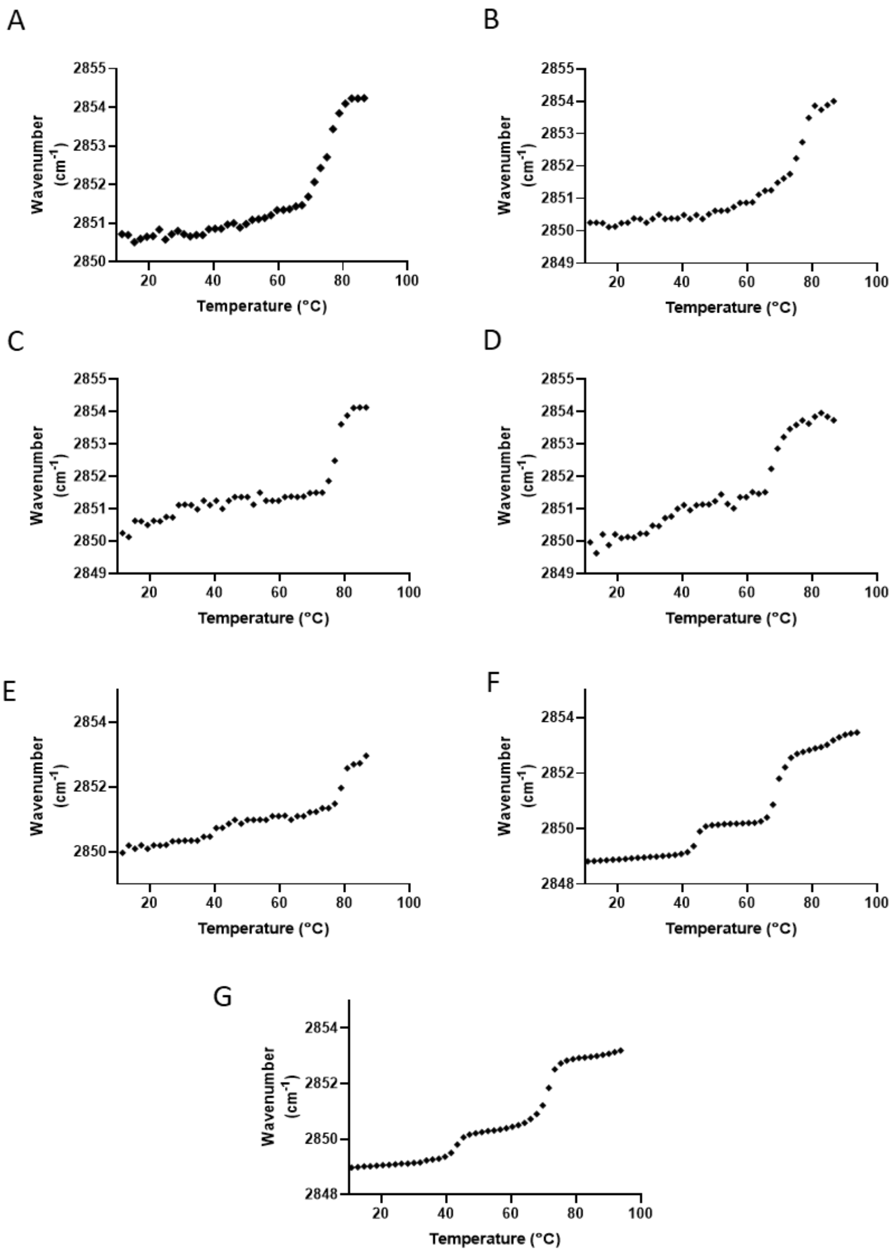

Figure S2: *Thermotropic response curves of the simple model with FFAs (A) C16, (B) C18 (C) C20 (D) C22 (E) C24 (F) C26 (G) C28. Error is calculated as the standard deviation. All models were prepared with CER:CHOL:FFA in a molar ratio of 1:1:1.*

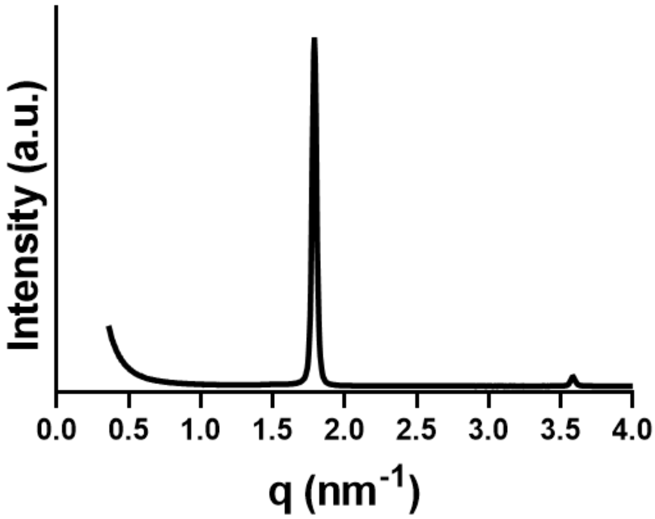

Figure S3 *X-ray diffraction curve of FFA C16, lipids form a bilayer with a repeat distance of 3.6 nm.*

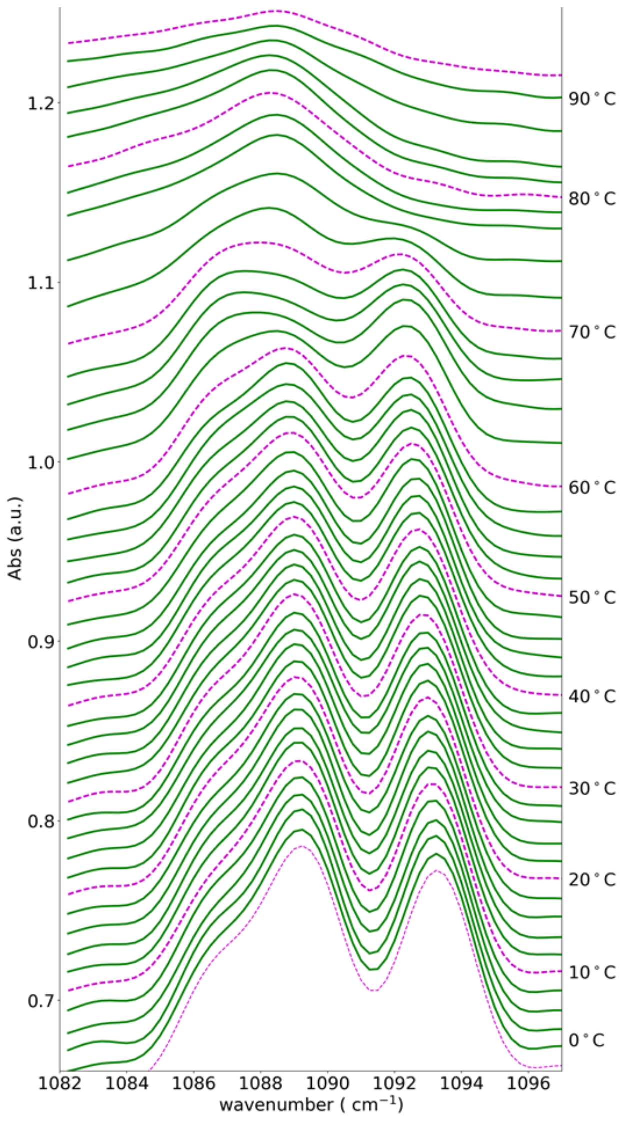

Figure S4: *δCD_2 of FFA C16 models, between 0 and 90 ºC. Every 10 ºC is highlighted by a dotted maroon curve. At low temperature the twin peaks or the orthorhombic phase at 1464 and 1475 cm^{-1}, can be observed with the single hexagonal peak at 1472 cm^{-1} These peaks remain as the temperature increases, until 76 ºC when the orthorhombic peaks are no longer present, and the lipids are melting. All models contain CERs EOS and NS at a ratio of 40:60. Overall the composition of CERs, CHOL and FFAs are in an equimolar ratio.*

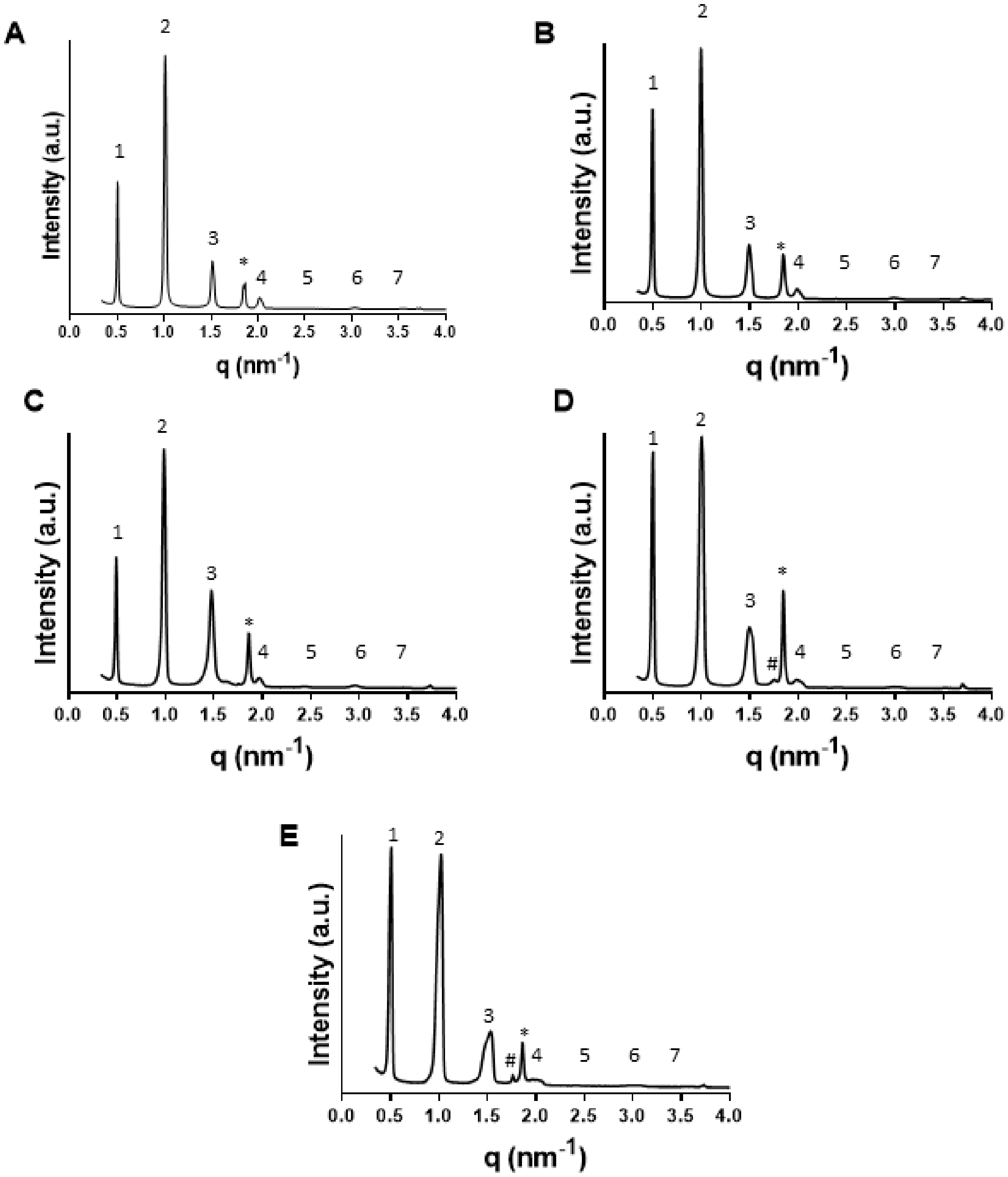

Figure S5 *SAXD curves for the porcine model containing FFA C16 at ratios of (A) 1.8, (B) 20, (C) 30, (D) 40, and (E) 50%. The Arabic numbers indicate the peak orders of the LPP. Crystalline CHOL peaks at 1.85 and 3.68 nm*$^{-1}$ *are indicated by an asterisk (*). The phase separation peak is indicated with a hashtag (#). All models were prepared with CER:CHOL:FFA in a molar ratio of 1:1:1.*

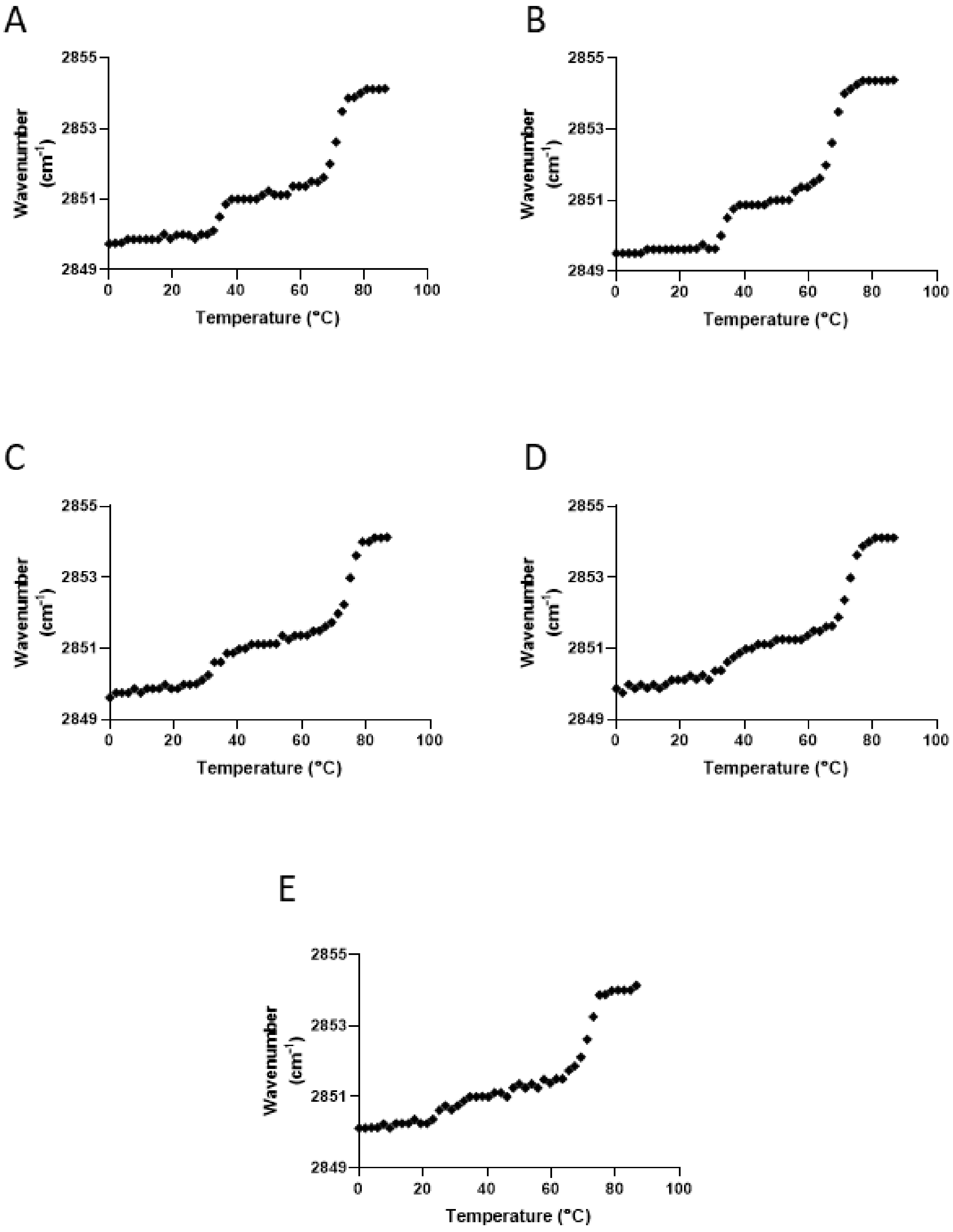

Figure S6 : *Thermotropic response curves of the porcine model with FFAs C16 content at (A) 1.8 (B) 20 (C) 30 (D) 40 and (E) 50% of total FFA content. Orthorhombic to hexagonal and hexagonal to fluid transition*

temperatures for all samples were similar with an average mid transition temperature of 32.7 ±1.6 and 71.0 ±2.2 ⁰C. All models were prepared with CER:CHOL:FFA in a molar ratio of 1:1:1.

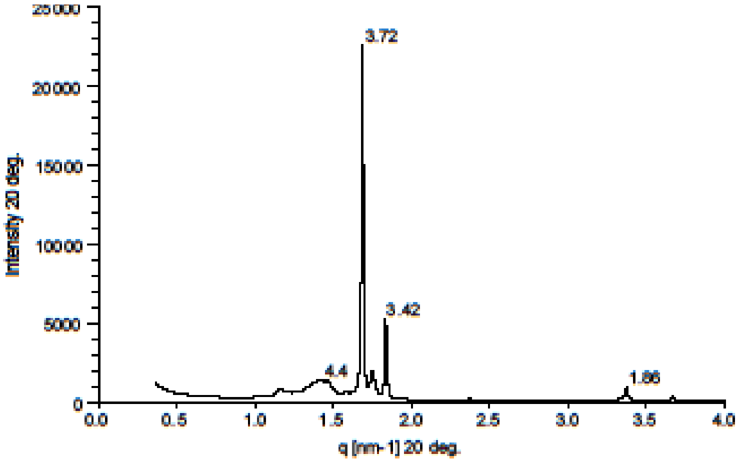

Figure S7 *Xray diffraction curve of CER NP: CHOL: FFA C16 at a 1:1:1 molar ratio, the main repeat distance is 3.7 nm, however additional structures at lower concentrations also forms at this composition.*
